# Optical Studies of Doped Two-Dimensional Lead Halide Perovskites: Evidence for Rashba-Split Branches in the Conduction Band

**DOI:** 10.1021/acsnano.4c01525

**Published:** 2024-07-01

**Authors:** Evan Lafalce, Rikard Bodin, Bryon W. Larson, Ji Hao, Md Azimul Haque, Uyen Huynh, Jeffrey L. Blackburn, Zeev Valy Vardeny

**Affiliations:** † Department of Physics and Astronomy, University of Utah, Salt Lake City, Utah 84112, United States; ‡ Chemistry and Nanoscience Center, National Renewable Energy Laboratory, Golden, Colorado 80401, United States; § Materials Science Center, National Renewable Energy Laboratory, Golden, Colorado 80401, United States

**Keywords:** perovskite, two-dimensional, doping, Rashba effect, infrared spectroscopy, Faraday rotation

## Abstract

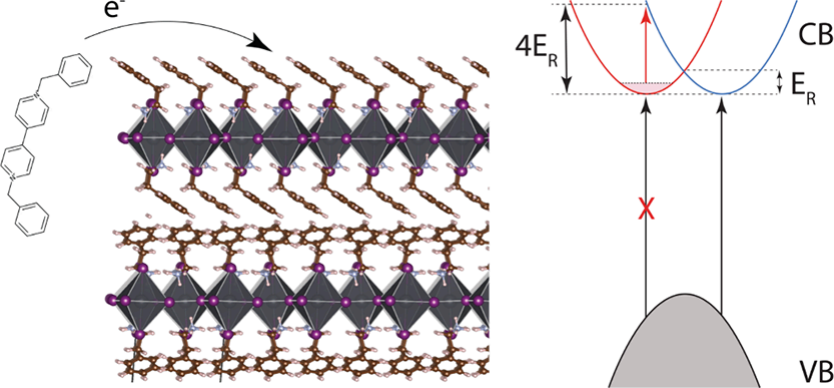

Two-dimensional (2D) hybrid organic/inorganic perovskites are an emerging materials class for optoelectronic and spintronic applications due to strong excitonic absorption and emission, large spin–orbit coupling, and Rashba spin-splitting effects. For many of the envisioned applications, tuning the majority charge carrier (electron or hole) concentration is desirable, but electronic doping of metal-halide perovskites has proven to be challenging. Here, we demonstrate electron injection into the lower-energy branch of the Rashba-split conduction band of 2D phenethylammonium lead iodide by means of n-type molecular doping at room temperature. The molecular dopant, benzyl viologen (BV), is shown to compensate adventitious p-type impurities and can lead to a tunable Fermi level above the conduction band minimum and increased conductivity in intrinsic samples. The doping-induced carrier concentration is monitored by the observation of free-carrier absorption and intraband optical transitions in the infrared spectral range. These optical measurements allow for an estimation of the Rashba splitting energy *E*_R_ ≈38 ± 4 meV. Photoinduced quantum beating measurements demonstrate that the excess electron density reduces the electron spin *g*-factor by ca. 6%. This work demonstrates controllable carrier concentrations in hybrid organic/inorganic perovskites and yields potential for room temperature spin control through the Rashba effect.

Hybrid organic/inorganic semiconductors (HOISs) with a perovskite crystal structure have been known for over 100 years but have attracted increasing attention in recent years as solution-processable semiconductors with exceptional optical and electronic properties that are on par with the best traditional semiconductors (e.g., GaAs). The broad applications envisioned for perovskite-based HOIS include photovoltaics,^1^ light-emitting diodes (LEDs),^2,3^ lasers,^4^ spintronic devices,^3,5,6^ and components of quantum information (QI) systems.^7,8^ Optoelectronic and spintronic devices based on traditional (e.g., III–V) semiconductors have been successful, in part, due to decades of research and development of methods to carefully tune and optimize the majority charge carrier type (electron or hole) and density. Yet, this level of control has yet to be achieved in most metal-halide perovskite semiconductors such as phenethylammonium lead iodide ((C_6_H_5_C_2_H_4_NH_3_)_2_PbI_4_ or PEPI).

Beyond the obvious impact on optoelectronic devices, controlled charge carrier doping can facilitate deeper understanding and manipulation of fundamental properties and dynamic processes in semiconductors. For example, doping of numerous low-dimensional semiconductors has been instrumental in identifying and exploiting multibody quasi-particles such as trions,^9−11^ three-body particles consisting of an exciton and an excess charge. Measuring changes to the electron spin *g*-factor in organic and inorganic semiconductors has elucidated mechanistic aspects of molecular redox doping,^12^ interfacial charge/spin transfer,^13^ and doping heterogeneity.^14^ For 2D HOISs such as PEPI, we hypothesized that the introduction of electrons into the conduction band of these materials by ground-state doping should facilitate the systematic investigation of Rashba splitting via infrared (IR) spectroscopy. Recently, Vardeny and co-workers utilized infrared photomodulation (PM) spectroscopy to demonstrate giant Rashba splitting in a polycrystalline 2D HOIS thin film.^15^ The study found photoinduced IR transitions that could be assigned to the transitions of either excitons or charge carriers between Rashba-split bands, which enabled estimation of the Rashba splitting energy, *E*_R_, of ca. 40 meV, an order of magnitude larger than observed for traditional III–V semiconductors.^16,17^ In contrast to the PM experiment, where an uncontrollable density of charge carriers presumably resulted from a small probability of excitons being autodissociated, ground-state doping should enable the careful injection of controlled densities of electrons into a 2D HOIS for studying Rashba splitting.

Although uncontrolled interactions with substrates^18^ and defects induced by off-stoichiometric precursor ratios^19^ have been shown to modulate the Fermi level and/or work function in HOIS, controlled electronic doping of HOIS remains a fundamental challenge.^20−22^ Electronic doping in semiconductors can be achieved by several means, among them two broadly used methodologies of substitutional aliovalent doping^23^ and molecular redox doping.^24^ Molecular redox doping relies upon the injection or removal of electrons from a semiconductor by a proximal molecule with either relatively low or high electron affinity, respectively. This technique has been used extensively in the organic semiconductor community to tune carrier type and density in, e.g., small molecules, polymers, and single-walled carbon nanotubes (SWCNTs).^25,26^ It has also been successfully applied to electronic doping of inorganic semiconductors, including transition metal dichalcogenides (TMDCs)^27^ and semiconductor nanocrystals (NCs).^28^ Recent studies have demonstrated that bulk,^29^ nanocrystalline,^24^ and 2D^30,31^ lead halide perovskite could be controllably doped p- or n-type by the adsorption of redox molecules on the perovskite surface.

In the current study, we employ the strong molecular reductant, benzyl viologen (BV), to demonstrate molecular n-type redox doping of PEPI. Optical absorption measurements in the visible and infrared regions of the spectrum are analyzed within a consistent framework that considers the effects of electron density and Fermi energy on the oscillator strength of both excitonic and free carrier transitions. The injected conduction band electrons induce broad infrared absorption features that are consistent with free carrier optical transitions between Rashba-split bands with a characteristic Rashba-splitting energy *E*_R_ ≈38 meV. Time-resolved Faraday rotation measurements demonstrate quantum beating of the spin-aligned electrons at ca. 2.4 eV. The electron spin *g*-factor, calculated from the quantum beating frequencies as a function of applied magnetic field, is reduced by ∼6% for n-type PEPI relative to undoped PEPI, consistent with the increased Fermi level expected for n-type doping. The results provided here elucidate a relatively simple strategy for electronic doping of 2D HOIS and for using excess charge carriers to both tune and quantify spin-related properties such as Rashba splitting.

## Results/Discussion

**Figure 1:**
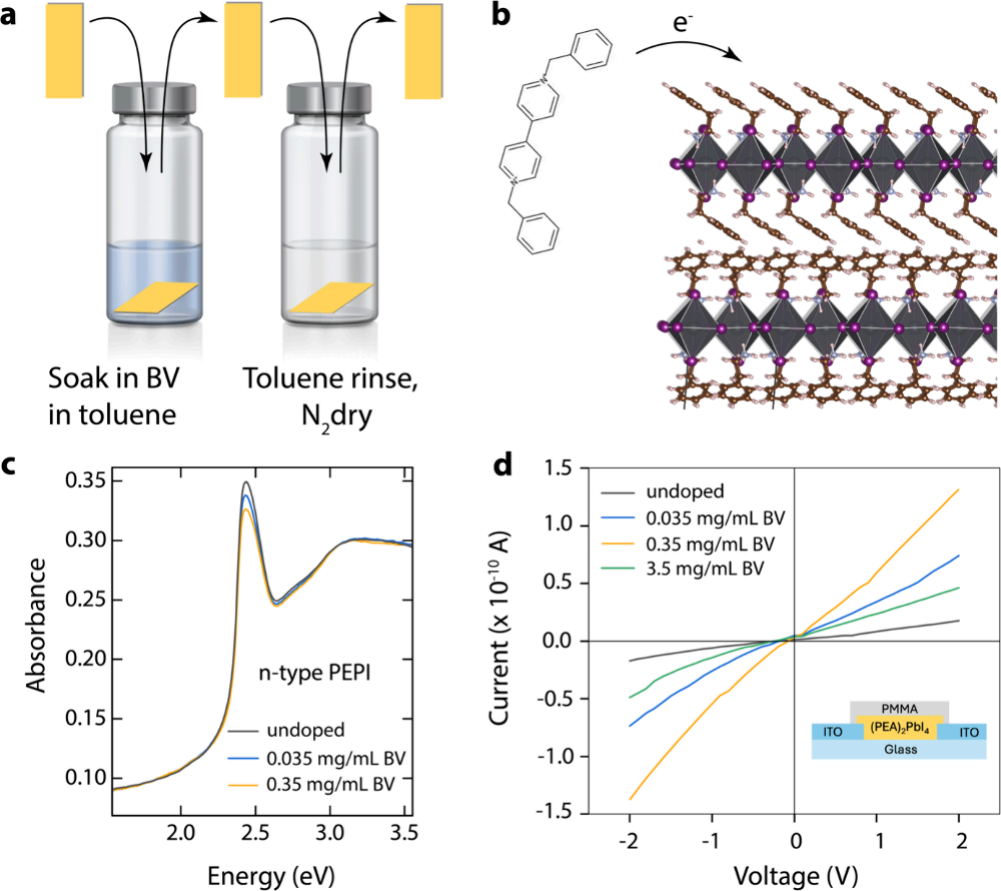
Doping of PEPI films with benzyl viologen (BV). (a) Schematic illustration of the doping process. The untreated PEPI film is soaked in a solution of BV in toluene for 60 s in an inert glovebox. The treated film is then rinsed with toluene to remove excess BV molecules from the surface of the film and blown dry with nitrogen gas (N_2_). (b) Schematic illustration of the donation of an electron from the BV molecule to the PEPI network. (c) UV/vis absorption of untreated films and films treated with dopant solutions with varying concentrations of BV. The exciton absorption band is increasingly quenched with increasing BV concentration. (d) Current–voltage (*I*–*V*) dependence for undoped and BV-doped PEPI thin films. Inset: schematic of the two-terminal architecture used for *I*–*V* measurements.

Figure 1a demonstrates the method of doping a PEPI thin film by exposure of the film to the molecular dopant benzyl viologen (BV) (synthesis of BV and PEPI, as well as film preparation details, can be found in the Methods/Experimental Section). Doubly reduced BV is prepared from the dichloride salt,^32^ resulting in a toluene solution of neutral BV with a concentration of 3.5 mg/mL that can be reduced by dilution. Soaking the PEPI film in this BV solution in a nitrogen glovebox allows for intercalation of the BV molecules into the film, with both dopant concentration and soaking time influencing the doping level. The low ionization potential of neutral BV makes it suitable to serve as an electron donor, and it is expected that exposure to BV will result in electrons being transferred (see Figure 1b) from the BV molecules to the PEPI lattice, rendering the film with an excess n-type carrier (electrons) concentration. The doped PEPI films are subsequently soaked briefly in toluene to remove excess BV molecules from the surface.

Figure 1c shows the absorption spectrum of a thin PEPI film formed by spin-coating on a quartz substrate before and after doping. The dominant feature at 2.4 eV arises from the optical transition of excitons with binding energy, *E*_B_, that has been estimated as *E*_B_ ≈250 meV.^33^ Higher valence band to conduction band transitions are also observed at 2.7 and 3.1 eV.^34^ Treatment with BV, which is expected to inject electrons into the PEPI conduction band, reduces the intensity of the exciton transition, and the transition intensity is inversely proportional to the concentration of BV used in the doping solution. These observations are consistent with the doping-induced reduction in oscillator strength experienced by semiconductors with sharp excitonic resonances, such as two-dimensional GaAs quantum wells,^35^ transition metal dichalcogenides,^36^ and semiconducting single-walled carbon nanotubes.^25^ The bleaching of the excitonic transition results from phase-space filling, where injection of either electrons or holes reduces the oscillator strength of an excitonic transition in a manner that is proportional to the injected carrier density.

To further confirm charge injection from BV into PEPI, we performed two-terminal conductance measurements (Figure 1d) on thin films of PEPI deposited across 200 μm channels between tin-doped indium oxide (ITO) contacts (Figure 1d, inset). Current–voltage measurements in Figure 1d demonstrate a clear increase in conductance (reflected by an increase in current at any given voltage) for PEPI films doped by 0.035–0.35 mg/mL BV, with the 0.35 mg/mL treatment increasing conductance by an order of magnitude. At the highest BV concentration of 3.5 mg/mL, the conductance decreases but remains higher than the undoped PEPI film. The decrease in conductivity at the highest doping level may be related to the formation of bipolarons that have lower mobility than free polarons, a well-known effect in molecular doping of π-conjugated polymers, a possibility that should be explored in future studies. These *I*–*V* measurements—and associated measurements in the Supporting Information (Figure S1)—confirm that the BV doping treatment injects electrons into the PEPI conduction band that increase the electrical conductivity.

**Figure 2:**
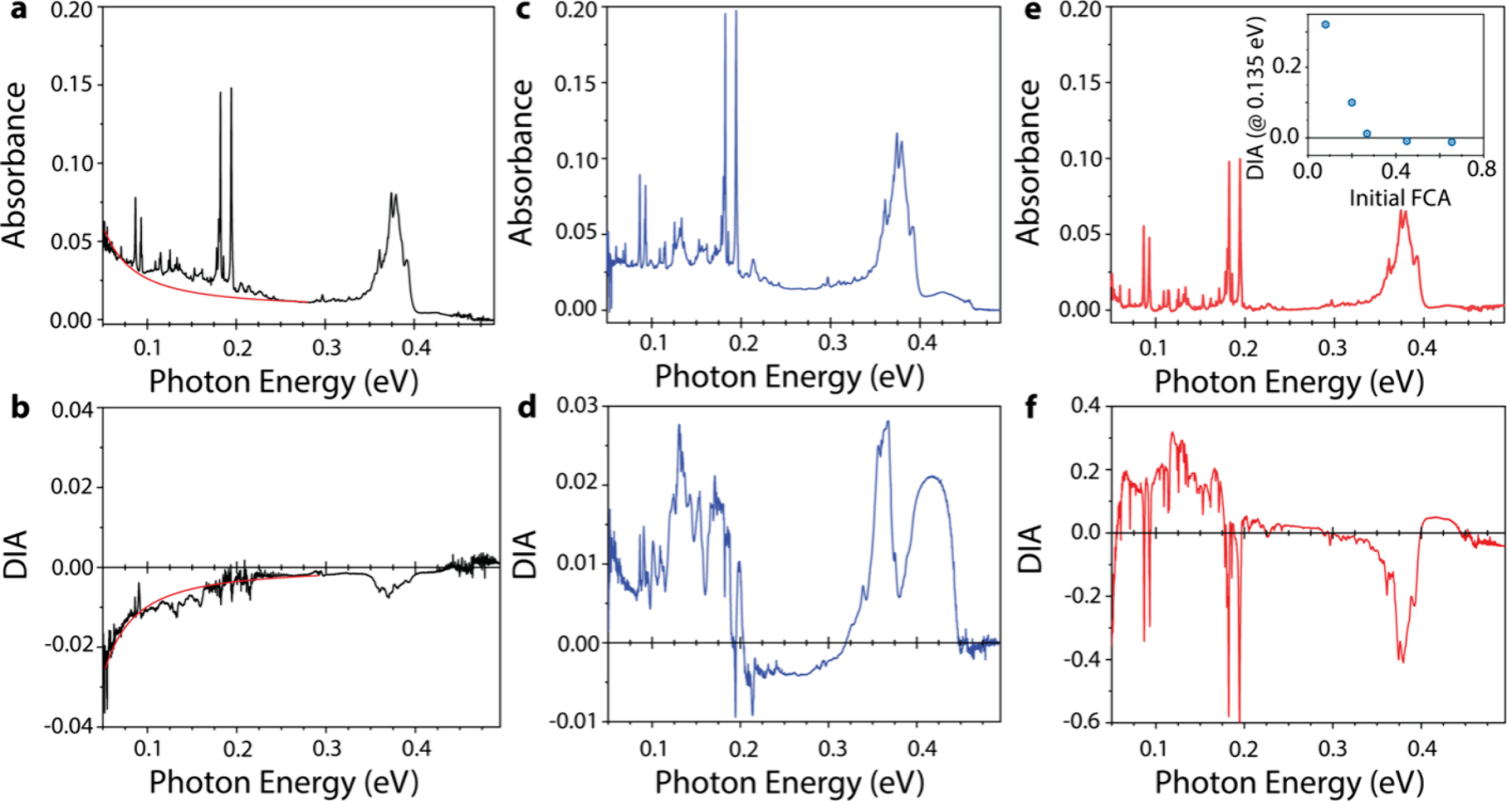
(a) FTIR absorbance of an untreated PEPI film that shows unintentional doping. (b) Doping induced absorption (DIA) of PEPI film in panel a doped with 3.5 mg/mL BV solution in toluene. Solid red lines in panels a and b are fits to a model for free-carrier absorption. (c, d; e, f) Same as in panels a and b but with two films with lower amounts of unintentional doping in the untreated films. The inset of panel e plots the DIA intensity in several PEPI films doped with 3.5 mg/mL BV solution in toluene vs the initial intensity of the free carrier absorbance (FCA, quantified as the ratio of the FCA at 0.05 eV to the absorbance strength of the vibrational mode at 0.38 eV), showing the inverse relationship between the observation of DIA in doped films and the observation of FCA in untreated films.

The effect of BV treatment was next characterized by Fourier transform infrared (FTIR) spectroscopy (Figure 2) in an attempt to observe IR transitions between Rashba-split electronic bands. The consistent IR features in Figure 2, across different films formed from nominally identical procedures, are a cluster of narrow absorption lines centered around 0.38 eV and strong peaks at 0.18, 0.19, 0.38 and 0.41 eV. All the peaks in this spectral range correspond to the vibrational modes of the organic PEA cations, whereas the vibrations of the inorganic octahedra occur at significantly lower energies.^37^ However, a difference between the three samples shown in Figure 2a,c,e is also apparent in the form of a slow rise in absorption at low energy. This band is attributed to free-carrier absorption (FCA) arising from electrons in the conduction band of PEPI samples. In Figure 2a we show a fit to a typical FCA semiconductor response, taken as *E*^–*p*^, where the exponent *p* depends on the dominant scattering mechanism for carriers in the conduction band. In this case, we determine from the fitting that *p* = 1.5, consistent with acoustic phonon scattering as the dominant relaxation mechanism of the carriers.^38,39^

The appearance of the FCA band in *untreated* PEPI films reveals adventitious impurity doping in PEPI films that results in moderate p-type (*vide infra*) doping. The presence of the band depends sensitively on the environmental conditions of the film fabrication, as a variation in the magnitude of this feature is observed across samples even when made in sequential order from the same solution. It is believed in this case that the vapor produced by the spin coating and subsequent annealing and crystallization of the PEPI films may cause the formation of an FCA band due to excess lead or other native defects that result in positively charged vacancies.

When the BV treatment described above is used to inject electrons into PEPI films, we observe a strong (inverse) correlation between the resulting doping-induced absorption (DIA) change and the strength of FCA in the untreated film. In the case of high levels of unintentional impurity doping (Figure 2a), the BV treatment results in a compensation effect, and the DIA shows a negative feature (Figure 2b) indicating a decrease in and bleaching of FCA. The bleach increases toward lower energy and can be fitted with an FCA model having an identical acoustic phonon scattering related power law as demonstrated in Figure 2b.

In contrast, as-cast films with low (Figure 2c) or absent (Figure 2e) impurity doping show positive DIA spectra (Figure 2d,f) after BV treatment. These DIA spectra have an “onset” at ∼0.22 eV and increasing intensity with decreasing energy, characteristic of intraband FCA, as well as an additional band centered near 0.135 eV. We attribute this additional band to transitions between levels in Rashba-split conduction bands based on prior IR photomodulation studies^34^ and additional studies described below. The strength of the DIA band is inversely correlated with the strength of the unintentional impurity doping generated FCA. We quantitatively demonstrate this anticorrelation in the inset of Figure 2e, where the intensity of the DIA band (at 0.12 eV) is plotted vs the ratio in the untreated film between the FCA at 0.05 eV to the absorption of the vibration at 0.38 eV. Consequently, we interpret the effect of the BV treatment as compensation of p-type unintentional impurity carriers or, in their absence, injection of carriers into the lower branch of the Rashba-split conduction bands.

**Figure 3:**
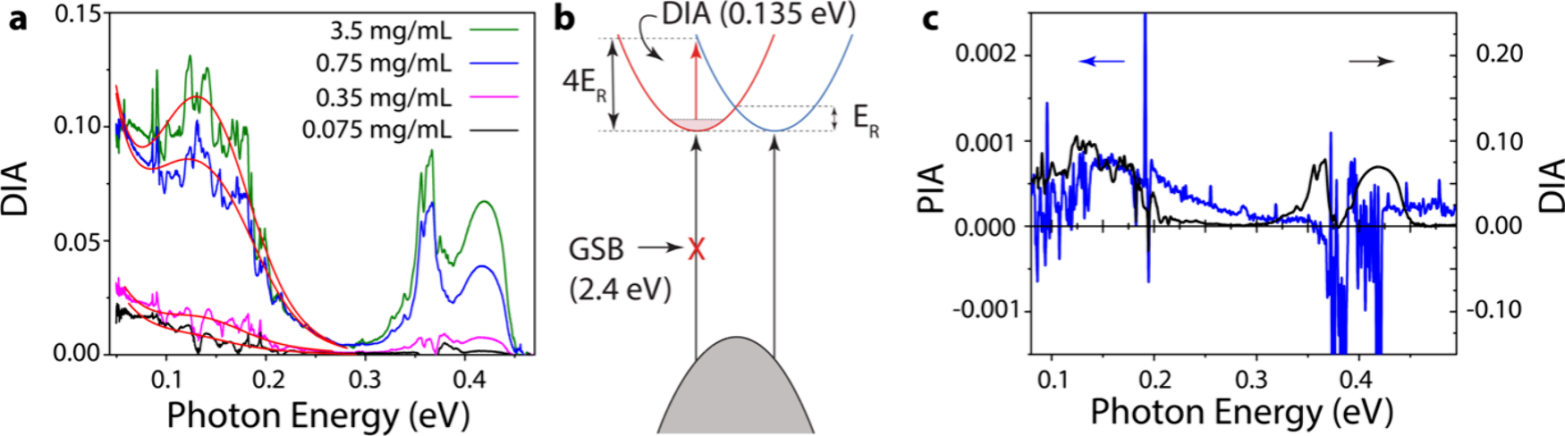
(a) DIA of a PEPI film for different concentrations of BV doping solution as indicated. Solid red lines are fits to the data according to the model described in the text. (b) Schematic of optically induced transitions in doped PEPI. BV doping introduces electrons and raises the Fermi level in the lower energy Rashba-split branch of the CB, enabling the intraband DIA spin-flip transition (red arrow). In the limit of low carrier density, the DIA transition is ∼4*E*_R_. The same CB electrons induce a ground-state bleach (GSB) of the excitonic transition, as seen in Figure 1c. (c) Comparison of photoinduced absorption (PA) of an untreated PEPI film (blue line) with DIA of a PEPI film doped with 3.5 mg/mL BV solution (black line), highlighting the similar induced absorptions observed in both experiments.

We now turn our focus to the DIA that results from BV treatment of PEPI samples with low/absent unintentional p-type impurity doping in the untreated state. In this case, the injected electron density is controllable via the concentration of BV molecules in the doping solution. As seen in Figure 3a, the observed n-type FCA and Rashba transition feature at ca. 0.135 eV both grow in intensity as the concentration of the BV doping solution increases. To support our assignment of the band centered at 0.135 eV to the “spin-flip” transition in the PEPI conduction band (Figure 3b), we consider the density of states (DOS) in Rashba-split conduction bands as a function of the carrier density-dependent Fermi level *E*_F_. In two dimensions, the DOS is constant in both branches of the conduction band. At low carrier density, the DIA peak would appear at 4*E*_R_, where *E*_R_ is the Rashba splitting energy (Figure 3b). As the injected carrier density increases, the band would broaden to both higher and lower energy as states at both higher and lower values of *k* away from the conduction band minimum become occupied, but the peak center will not shift (Figure S2).

To account for the occupation of states in the lower branch of the conduction band in the presence of thermal broadening, the absorption features due to the interbranch transitions, DIA_R_, are represented by a product of two Fermi–Dirac distributions:
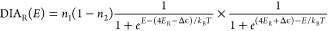
1where *n*_1_ and *n*_2_ are the density of electrons in the lower and upper branches, respectively, and Δϵ is the spread in the energetic transition energies. The solid red lines in Figure 3a shows the result of the fitting to a sum of the FCA component and DIA_R_, i.e. *A*(*E*) = *aE*^1.5^ + *b*DIA_R_, where *a* and *b* are the relative amplitudes of the FCA and interbranch transition contributions, respectively, to the spectra. After extracting the value of *E*_R_ from the peak position, the model may be used to obtain the values of the Fermi level *E*_F_ from the energetic broadening Δϵ. Based on the model, Δϵ is determined by *E*_F_ through the broader range of available transitions between the sub-branches of the conduction band with increased occupation of the levels in the lower branch. Table 1 shows the parameters extracted from fitting the data in Figure 3a to eq 1 for different dopant concentrations. Both the FCA and the interbranch transition amplitudes increase with increased concentration, although the strength of FCA tends to saturate at higher carrier densities.

**Table 1:** Extracted Values of the Relative Weights of the DIA_R_ and FCA Terms and Fermi Level *E*_F_ from the Fit of the DIA Spectra of PEPI Films Doped with Different Concentrations of BV Solutions in Toluene^a^

dopant concentration	*a*	*b*	*E*_F_ (meV)
0.075 mg/mL	3.6 × 10^–4^	0.008	0.7
0.35 mg/mL	4.1 × 10^–4^	0.017	2.1
0.75 mg/mL	1.1 × 10^–3^	0.088	4.6
3.5 mg/mL	1.2 × 10^–3^	0.136	4.7

a *E*_F_ is extracted from eq 1 and is referenced to the bottom of the conduction band.

The model discussed above describes our data most effectively for a Rashba splitting energy *E*_R_ = 37.5 meV. A similar feature has been observed in the photoinduced absorption spectrum due to photogeneration of carriers in the undoped PEPI film,^34^ as shown in Figure 3c. The peak centers from the two measurements are similar, and we obtain a value of *E*_*R*_ = 40 ± 5 meV from the photomodulation (PM) measurement. The successful application of the simple model described here to track both the FCA and DIA response of BV-treated PEPI supports the conclusion that the BV treatment successfully injects electrons into the PEPI conduction band. Furthermore, the near-identical values of *E*_R_ extracted from the DIA and PM measurements demonstrate that the redox doping methods developed here represent a relatively simple strategy for probing Rashba splitting in HOIS. The comparison in Figure 3c also allows us to comment on the debate over the presence of polarons in perovskites. In fact, it has been suggested that the PA results displayed in Figure 3c may not distinguish between polaron absorption bands or those due to Rashba-split conduction band interbranch transitions.^34^ In DIA, however, we simultaneously observe both FCA and the Rashba interbranch transition. This observation is inconsistent with the formation of small polarons, as the transition between small polarons levels and the continuum would be blocked by the presence of the free carrier density.

Additional information can be gleaned from the FTIR spectra of the doped PEPI films, which supports our conclusion that electrons are successfully injected into PEPI by adsorbed BV molecules. For example, a rich spectrum of observed Fano resonances likely results from coupling of the narrow vibrational transitions to the broad FCA continuum, as observed for, e.g., doped SWCNTs.^40,41^ At higher energy, the cluster of vibrational peaks in pristine PEPI is modulated, resulting in a derivative-like feature as well as some absorption from the adsorbed BV dopant molecules on the surface (Figure S3). In the visible/near-infrared spectral range, doping-induced absorption is also observed at energies just below the exciton absorption, indicating transitions from the valence band into ionized dopant levels energetically positioned in the gap close to the conduction band (Figure S4).

With electron-doped PEPI in hand, we may also probe the degree to which excess electrons in the CB can modulate the spin- and optoelectronic properties of this prototypical 2D HOIS. Here, we turned to picosecond (ps) time-resolved Faraday rotation (TRFR) spectroscopy, a pump–probe experiment that can be used to measure the characteristic electron *g*-factor (*g*) in photoexcited semiconductors. The pump beam is circularly polarized and generates spin polarized electrons. These electrons begin to precess about an applied magnetic field *B*, in the Voigt configuration, with a frequency (ω) described by Larmor spin precession:
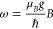
2

In equation 2, *μ*_B_ is the Borh magneton, or the magnetic moment of an electron, and ℏ is the reduced Planck's constant. The linearly polarized probe pulse interacts with the electron spin as a rotation of the plane of polarization, Faraday rotation, which is measured at the photodetector. The oscillation in the signal resulting from the precession of spin polarized electrons is known as quantum beating (QB). By comparing the QB dynamics in pristine and doped PEPI, we are able to follow the *g*-value change as the doping induced Fermi level increases in the CB.

**Figure 4:**
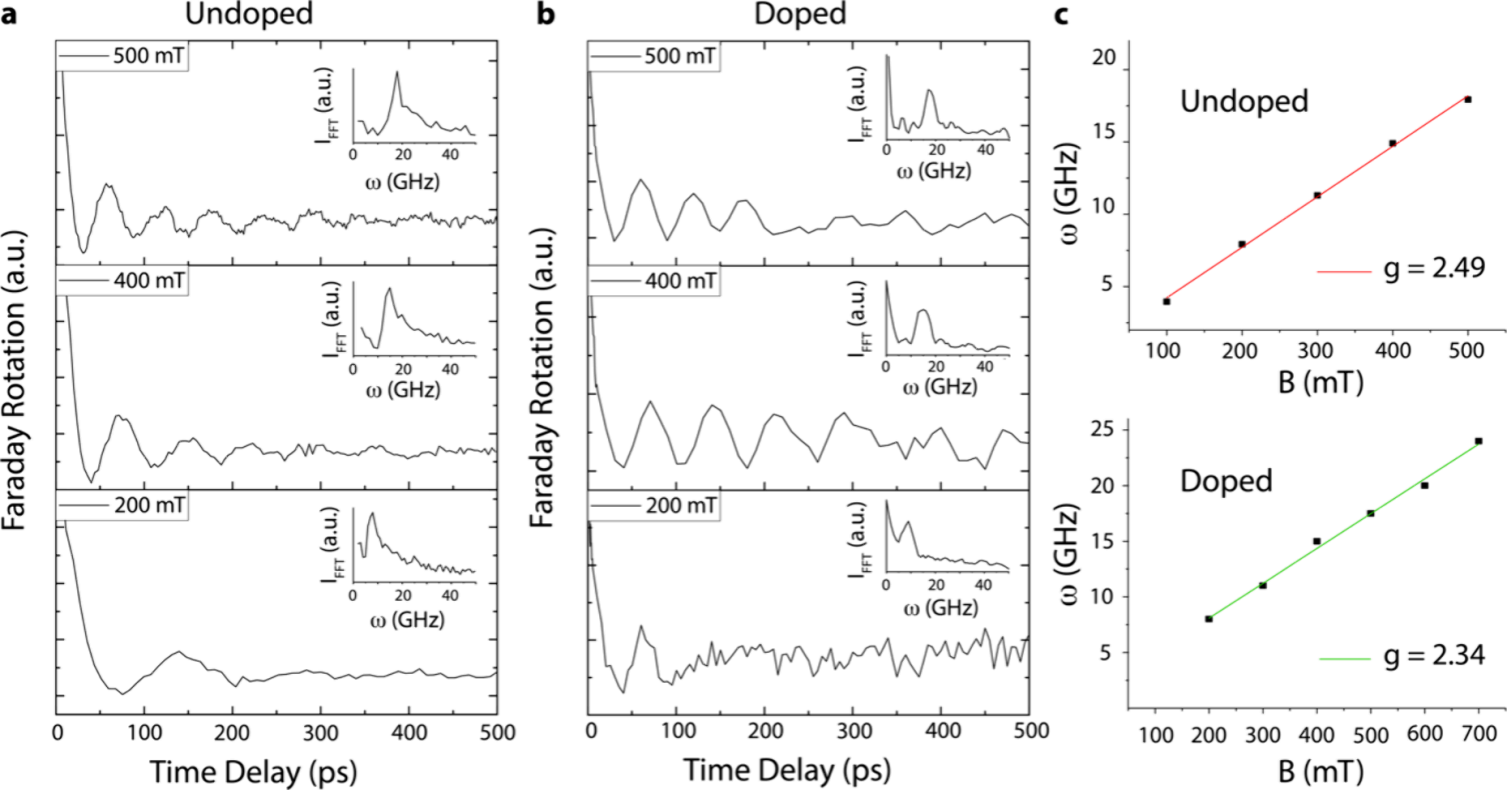
Time-resolved Faraday rotation (TRFR) response of a pristine (a) and doped (b) 2D PEPI film measured at three different magnetic field strengths in the Voight configuration. The related FFT spectra are shown in the appropriate panels and used to extract the QB frequencies. (c) Plots of the QB frequency vs the applied magnetic field *B*, which are used to determine the Lande *g*-factor for the pristine (lower panel) and doped (upper panel) films via the Larmor spin precession formula (eq 2).

The TRFR responses of pristine and doped PEPI are shown in Figure 4a,b, respectively. Quantum beating can be readily observed in both samples (main panels), and fast Fourier transform (FFT) spectra (insets) are used to extract the Larmor oscillation frequencies. The plot of the QB frequency (ω) versus *B* is a linear relationship (Figure 4c) described by eq 2. Extracting the *g*-factors of the doped and undoped samples with eq 2 reveals a decrease in the electron *g*-factor with doping, i.e., from *g* ≈2.49 ± 0.06 (pristine undoped) to *g* ≈2.32 ± 0.12 (n-type). This change can be attributed to the doping-induced change in the Fermi level, whereby the increased electron energy and k-vector in reciprocal space cause a decrease in the electron *g*-value.^42^ These results can be also viewed as additional evidence that successful n-type doping into PEPI has occurred.

The results of this study provide an experimental framework for modulating carrier density in 2D HOIS via molecular doping and also for using the excess injected carriers to probe fundamental spin-related properties such as Rashba splitting and the electron *g*-value in the conduction band. The Rashba effect, a momentum-dependent splitting of spin bands within a crystal, is recognized as a powerful material property for the manipulation of spins with external stimuli (e.g., electric fields, magnetic fields, etc.). Rashba splitting occurs in the presence of SOC but also requires the breaking of inversion symmetry. A number of recent theoretical and experimental studies have found evidence for Rashba splitting in HOIS,^43−50^ and it is thought that Rashba splitting may be linked to a number of emergent phenomena in HOIS, including long spin-coherence times,^47,51^ low recombination rates,^48,52^ spin-charge conversion,^53,54^ and high photoluminescence yields.^50^ However, the precise crystallographic, structural, and electronic origin of strong Rashba splitting in HOIS is still unclear, and the community is still developing ways to rapidly screen materials for Rashba splitting in the conduction and valence bands. The relatively simple methodology described here provides a rapid experimental screen, whereby doping-induced infrared transitions can provide evidence for Rashba splitting in HOIS.

From a broader standpoint, our study demonstrates that molecular redox doping is a viable strategy for modulating carrier density and type in 2D metal halide perovskite semiconductors. There are still relatively sparse reports on successful electronic doping of 2D HOIS despite the potential technological impacts that this level of control could facilitate. The n-type doping demonstrated here complements the recent demonstration of p-type doping of 2D perovskites,^30,31^ outlining a path toward controlling optoelectronic properties in this important class of hybrid semiconductors.

## Conclusions

Molecular doping of 2D perovskite PEPI by the molecule benzyl viologen reveals exciton quenching, increase of electron density, and increased electrical conductivity. Both free-carrier absorption and transitions between the lower branch and the upper branch of the Rashba split conduction band were observed as a result of the chemically driven electron injection. The doping induced absorption band was comparable to the photoinduced absorption band, providing an independent confirmation of Rashba splitting in PEPI with a Rashba energy of ∼38 meV. The doping induced absorption features increased with increasing concentration of the dopant solution, allowing for control of the Fermi level inside the conduction band of the perovskite semiconductor. The realization of controllable carrier densities in Rashba split semiconductors may provide a significant step toward tunable functionalities in perovskite-based spintronics and optoelectronic devices.

## Methods/Experimental Section

### PEPI Synthesis/Deposition

Phenylethylammonium iodide ((C_6_H_5_C_2_H_4_NH_3_)I) was combined in a 2-to-1 molar ratio with lead iodide (PbI_2_) in dimethylformamide (DMF) to form (C_6_H_5_C_2_H_4_NH_3_)_2_PbI_4_ in a 0.2 M solution in DMF. The solution was subsequently stirred for 24 h on a hot plate held at 50 °C. Films were prepared on KBr or quartz substrates by spin-coating at 2300 rpm for 90 s and subsequently annealed for 15 min on a hot plate at 90 °C. This procedure resulted in films of 270 ±40 nm thickness.

### Benzyl Viologen Preparation and Film Doping

Benzyl viologen (BV) was prepared as the doubly reduced neutral molecule by reduction of the BV cation in the benzyl viologen dichloride salt. In a 20 mL borosilicate vial, 35 mg of benzyl viologen dichloride was dissolved in 10 mL deionized water. Upon full dissolution of the salt, 10 mL of toluene was layered on top of this aqueous solution. The biphasic solution was then sealed with a septum, and nitrogen was bubbled vigorously through the solution for 10–15 min to purge the solution of oxygen. In a separate vial, 200 mg of NaBH_4_ was added to 1 mL of deionized water, which initiates vigorous bubbling. After allowing this NaBH_4_ solution to evolve gas for approximately 2 min, the solution was drawn into a syringe and injected into the benzyl viologen dichloride solution (with N_2_ gas still bubbling through the biphasic solution). Two-electron reduction of the viologen salt with NaBH_4_ turns the BV from clear to blue/violet and causes the neutral BV to migrate into the toluene phase. The reaction is allowed to progress under constant nitrogen bubbling for 10–20 min. The dark blue/violet toluene layer containing BV is then drawn into a 20 mL syringe and transferred to a sealed and nitrogen-purged vial that is subsequently pumped into the glovebox. The 3.5 mg/mL stock solution prepared in this manner is used as a source for a series dilution resulting in 0.35, 0.035, and 0.0035 mg/mL solutions in toluene. PEPI films were doped by immersion in benzyl viologen (BV) (n-type doping) solution in toluene for 60 s. After immersion, the samples were rinsed for 1 s in toluene and blown dry with N_2_ gas.

### Electrical Conductance Measurements

ITO-coated glass substrate was patterned by using hydrochloric acid and zinc metal powder to create channels for electrical measurements. PEPI was deposited on the ITO substrates by spin coating as described previously. Finally, PMMA in chlorobenzene was spin coated on the device to avoid any influence of ambient conditions on the electrical measurements. Electrical characterization was carried out using a Keithley 4200 semiconductor analyzer connected to a Signatone probe station.

### Fourier Transform Infrared Spectroscopy

Fourier transform infrared (FTIR) absorption measurements were performed using a Thermo Scientific Nicolet iS-50 spectrometer equipped with a KBr beam splitter and a DLaGTS detector. During the measurement, the samples were housed in an optical cryostat with KBr windows and held in dynamic vacuum at a pressure of 50 mTorr. Absorption measurements in the visible/near-infrared spectral range were performed using a Cary spectrophotometer in ambient conditions. Doping induced absorption (DIA) is obtained as the difference between absorption prior to and subsequent to immersion in the dopant solution.

### Time-Resolved Faraday Rotation (TRFR) Measurements

Quantum beatings (QBs) were measured using time-resolved Faraday rotation (TRFR), a variant of the pump–probe experiment. Two pulses of light, ∼ 150 fs pulse duration and 80 MHz rep rate, were generated using a Ti/sapphire laser (Spectra Physics 3955 Model). The output of the Ti/sapphire laser was combined with the infrared output of an optical parametric oscillator (OPO) in a BBO type 2 sum frequency generation (SFG) crystal to generate the probe pulse at 526 nm.. The pump pulse, 405 nm, was generated with the fundamental in a second harmonic generation BBO crystal. The linearly polarized probe was modulated via a chopper, whereas the circularly polarized pump was modulated from left to right-handed via a P.E.M. The sample was housed in a closed loop helium cryostat at 4 K with a built-in magnet capable of producing magnetic fields up to 700 mT. The signal resulted from the transmission of the probe pulse after being directed through a beam splitter and into a balanced photodetector. The detector was connected to two lock-in amplifiers; the first one was synchronized to the chopper at 1.2 kHz, and the second was synced to the P.E.M. at 41 kHz. A mechanical delay stage was used to delay the probe pulse from the pump pulse, accessing temporal resolution.

## Supporting Information Available

Additional electrical transport measurements; infrared transitions of electrons in Rashba-split conduction band; FTIR absorption of BV on KBr; and visible to near-infrared absorption of a PEPI film before and after BV treatment (PDF)
