# Supplementary material for: Optical Studies of Doped Two-Dimensional Lead Halide Perovskites: Evidence for Rashba-Split Branches in the Conduction Band
Source: ACS Nano. 2024 Jul 1;18(28):18299–306. doi: 10.1021/acsnano.4c01525 (PMC11256896; doi:10.1021/acsnano.4c01525)
Supplement: Supplementary file 1 — nn4c01525_si_001.pdf [file nn4c01525_si_001.pdf]

Optical Studies of Doped Two-dimensional Lead Halide Perovskites: Evidence for Rashba-Split Branches in the Conduction Band

Evan Lafalce,[1] Rikard Bodin,[1]* Bryon W. Larson,[2] Ji Hao,[3] Md Azimul Haque,[3] Uyen Huynh,[1] Jeffrey L. Blackburn,[3]* Zeev Valy Vardeny,[1]**

1. *University of Utah, Department of Physics and Astronomy, Salt Lake City, UT 84112, United States.*
2. *Chemistry and Nanoscience Center, National Renewable Energy Laboratory, Golden, CO 80401, United States.*
3. *Materials Science Center, National Renewable Energy Laboratory, Golden, CO 80401, United States.*

Supplementary Information

1. Additional Electrical Transport Measurements

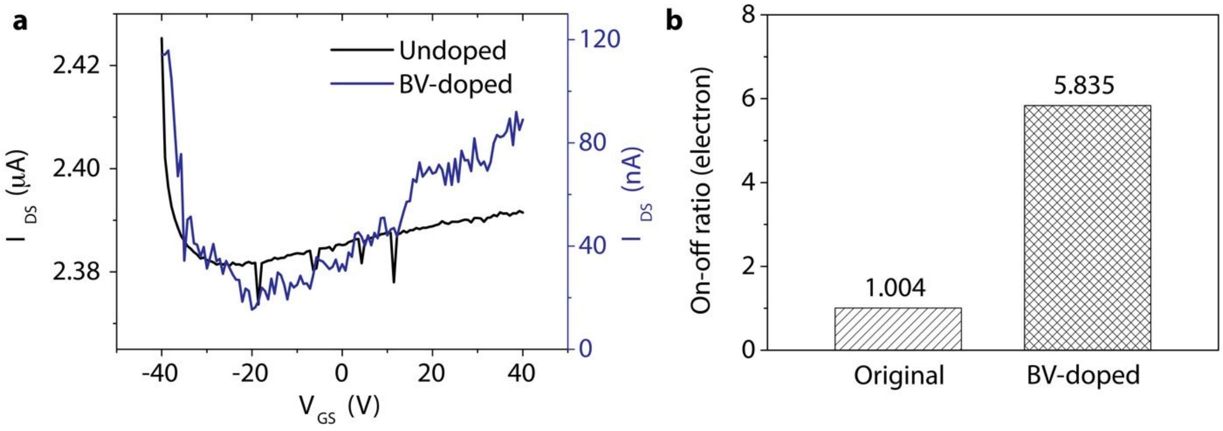

Figure S1. (a) Source-drain current (I_{DS}) vs. gate voltage (V_{GS}) of PEPI based FET before and after BV doping. (b) On-Off ratio for undoped and BV doped PEPI FET.

To characterize the impact of injected electrons on electrical transport in PEPI thin films, we performed additional field-effect transistor (FET) measurements. Our current FET capabilities are developed primarily for bottom-gated and bottom-contacted devices where the source-drain contacts are patterned gold electrodes. All FET devices were fabricated by utilizing standard optical lithography in the cleanroom. The devices consisted of a p-doped silicon wafer with a 200-nm thickness SiO_2 layer, with electrodes deposited as a 5-nm Ti adhesion layer and an 80-nm Au layer. For deposition onto FET substrates, the area outside of the channel was first masked with Kapton tape. PEPI precursor solution was spin-cast onto the FET channel and the substrate edges were then carefully wiped with a q-tip to remove excess PEPI and leave just the FET channels covered. FET transfer curves, **Figure S1**, were measured before and after doping.

Before doping, the PEPI FETs are ambipolar with varying on/off ratios for both the p- and n-channels, though all have substantial p-type character and weak or no n-type character. As such, it is important to

compare the exact same deposited PEPI layer, and doping is performed on the FET chip after running the sample in its undoped state. Doping with BV increases the on/off ratio of the n-channel by six-fold relative to the same film in its undoped state. These changes are consistent with BV injecting electrons into the conduction band, as expected, and as observed previously for a number of different organic and inorganic semiconductors.[2-4] Interestingly, although BV molecules appear to inject the appropriate charge carriers, the conductivity decreases for the doping treatment. Importantly, we note that the on- and off-currents for both undoped and BV-doped FETs are orders of magnitude larger than the leakage current, which is in the range of ca. 1 nA. The source of the conductivity reduction in the BV-doped FET is currently unclear, although it may result from a number of phenomena, including: an increased Schottky barrier at the interface between gold and n-type PEPI since gold has a relatively high work function (ca. 5.6 eV); physical deterioration of the n-type PEPI/gold interface from the solution doping treatment; deleterious ion migration and screening of the field in the n-type FET due to the large source-drain bias required to gate the device.

2. Infrared Transitions of Electrons in Rashba-split Conduction Band

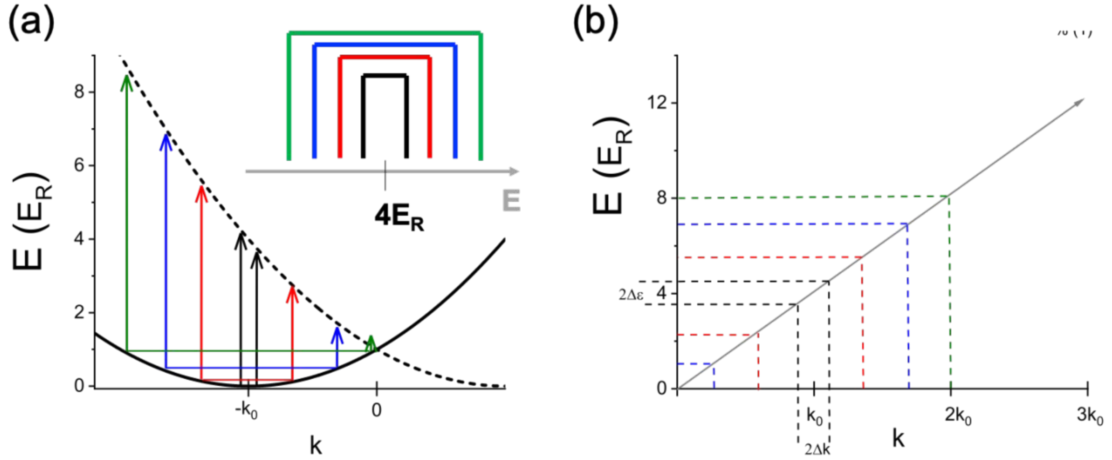

Figure S2. (a) Schematic diagram of transitions between branches of the conduction band in the presence of Rashba splitting with increasing Fermi level. The inset shows the expected line shape at absolute zero temperature. (b) Energy of transitions vs. k in units of the Rashba energy, E_R. The dashed lines indicate the bounds of the spread in transition energies $\Delta\epsilon$ and the related spread in occupied states in k-space Δk with increasing Fermi level.

We consider the infrared optical transitions induced by presence of carriers injected into Rashba split conduction bands. The transitions as a function of doping-induced Fermi level, E_F, are illustrated in **Figure S2a**. For a single electron in the lower conduction band, we expect an optical transition to occur at $4E_R$ of infinitesimal width. As more carriers are injected into the band and E_F increases, transitions at both higher and energy lower energy than $4E_R$ become available. Through the transition energy,

$$E(k) = {}^{\hbar^2}\!/_{2m}(k_0 + k)^2 - {}^{\hbar^2}\!/_{2m}(k_0 - k)^2 = {}^{\hbar^2}\!/_{2m}(4k_0 k)$$

A linear relation exists between the spread in k of occupied states, $2\Delta k$, and the spread in transition energies available, $2\Delta\epsilon$ as shown in **Figure S2b**. To derive an appropriate lineshape function of the infrared absorption, we need to consider the joint density of states. Because the electronic system is two-dimensional (2D), the density of states of the bands are constant. The joint density of states then directly mirrors the Fermi-Dirac distribution of occupied and unoccupied states in the lower and upper Rashba-split conduction bands, respectively. We thus expect a box-shaped absorption band centered at $4E_R$ that broadens with increasing Fermi level. We model this by a product of Fermi-Dirac functions,

$$DIA_R(E) = \frac{n_1}{1 + e^{\frac{E - (4E_R - \Delta\epsilon)}{k_B T}}} \times \frac{1 - n_2}{1 + e^{\frac{(4E_R + \Delta\epsilon) - E}{k_B T}}}$$

Where n_1 and n_2 are the densities of carriers in the lower and upper branches respectively, E_R is the Rashba energy, and $k_B T$ is the thermal energy available to the doped electron system. Furthermore, we can determine the Fermi level from the parameter $\Delta\epsilon$ since

$$E_F = {}^{\hbar^2}\!/_{2m}(\Delta k)^2 = {}^{\hbar^2}\!/_{2m}\left(\frac{m k_0 \Delta\epsilon}{2\hbar^2}\right)^2 = \frac{\Delta\epsilon^2}{16E_R}$$

As long as $E_F \leq E_R$.

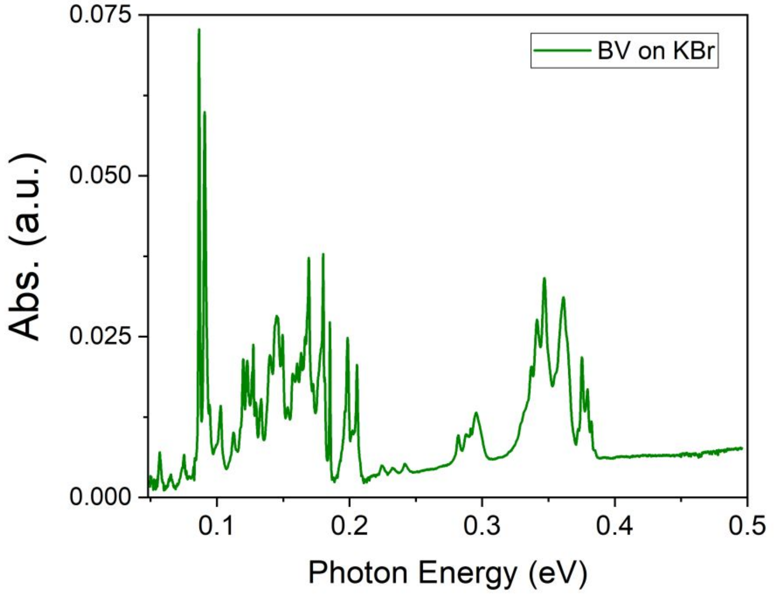

Figure S3. FTIR absorption of BV on KBr.

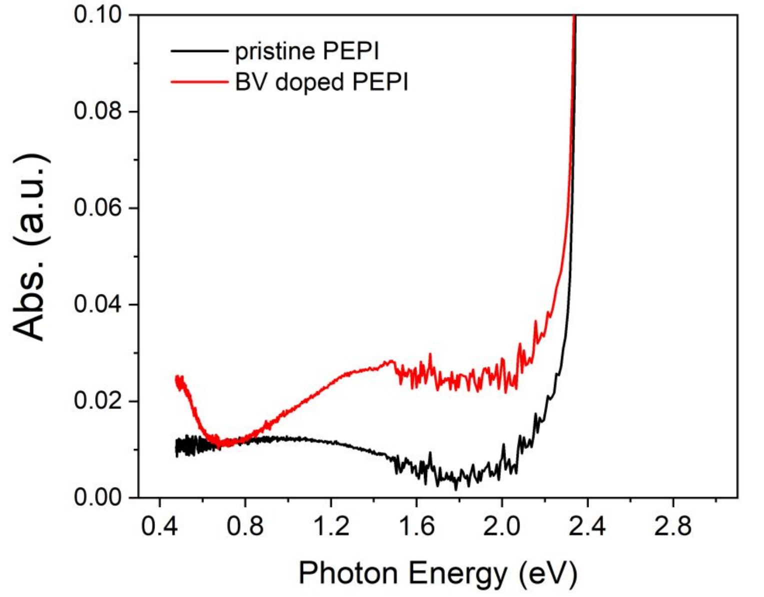

Figure S4. Visible to near-infrared absorption of a PEPI film before (black) and after (red) BV treatment.
